# Multilevel Analysis of the Influence of Maternal Smoking and Alcohol Consumption on the Facial Shape of English Adolescents †

**DOI:** 10.3390/jimaging6050034

**Published:** 2020-05-18

**Authors:** Jennifer Galloway, Damian J.J. Farnell, Stephen Richmond, Alexei I. Zhurov

**Affiliations:** School of Dentistry, Cardiff University, Heath Park, Cardiff CF14 4XY, UK; FarnellD@cardiff.ac.uk (D.J.J.F.); RichmondS@cardiff.ac.uk (S.R.); ZhurovAI@cardiff.ac.uk (A.I.Z.)

**Keywords:** smoking, alcohol, facial shape, multilevel PCA, ALSPAC

## Abstract

This cross-sectional study aims to assess the influence of maternal smoking and alcohol consumption during pregnancy on the facial shape of non-syndromic English adolescents and demonstrate the potential benefits of using multilevel principal component analysis (mPCA). A cohort of 3755 non-syndromic 15-year-olds from the Avon Longitudinal Study of Parents and Children (ALSPAC), England, were included. Maternal smoking and alcohol consumption during the 1st and 2nd trimesters of pregnancy were determined via questionnaire at 18 weeks gestation. 21 facial landmarks, used as a proxy for the main facial features, were manually plotted onto 3D facial scans of the participants. The effect of maternal smoking and maternal alcohol consumption (average 1–2 glasses per week) was minimal, with 0.66% and 0.48% of the variation in the 21 landmarks of non-syndromic offspring explained, respectively. This study provides a further example of mPCA being used effectively as a descriptive analysis in facial shape research. This is the first example of mPCA being extended to four levels to assess the influence of environmental factors. Further work on the influence of high/low levels of smoking and alcohol and providing inferential evidence is required.

## 1. Introduction

In facial shape research, we are faced with a large number of outcome variables in the form of facial landmarks. Principal component analysis (PCA) is a well-established technique for dimension reduction and can be used to help make analyses more manageable. However, conventional PCA calculates the principal components (PCs) and their corresponding eigenvalues using the dataset as whole, which ignores any groups or clusters that occur naturally in the population of subjects or shapes [1]. Such clusters often occur due to factors/covariates such as sex, age, or ethnicity. Furthermore, we cannot prevent the effects of different factors/covariates becoming “mixed together” in conventional PCA because PCs are orthogonal to each other. Further, the effects of some covariates on facial shape might be small and conventional PCA therefore limits our ability to assess subtle differences in facial shape because this might be “lost in the tail” of the eigenvalues. Halazonetis [2] points out yet another potential limitation of conventional PCA, stating: “A significant disadvantage of PCA is that the resulting components, because they are derived statistically, do not necessarily have a clear biological interpretation. Usually, only the first, or the first few, can be described satisfactorily...” (p. 580). 

We believe taking a multilevel approach to PCA is an elegant way to overcome these issues and can be seen as a non-trivial extension to PCA. Multilevel PCA (mPCA) addresses these problems directly by representing (and so isolating to some extent) specific effects at separate levels of the model. Furthermore, although PCs are still orthogonal within each level using mPCA, they do not have to be orthogonal between levels. This process should, in principle, reduce the amount of mixing due to orthogonality in conventional PCA. Therefore, mPCA allows dimension reduction to be carried out in a supervised manner, thus increasing our confidence that the information we retain within the level-specific PCs describes our covariates of interest. mPCA has been described by Lecron et al. [3] and used in the segmentation of spine radiographs. Subsequently, mPCA has been used to assess dental radiographs [4] and has been successful in assessing the influence of various covariates on facial shape, including ethnicity, sex, smiling and age [5,6,7,8,9]. These papers document the progression of the mPCA technique and provide the mathematical background. 

mPCA gives a number of benefits. Firstly, we are able to calculate the percentage of the total variation due to each covariate. This provides useful information and allows us to determine the importance of certain covariates in facial shape research. Secondly, mPCA allows us to be more confident that differences between groups are due to our covariate of interest and allows the separation of the influence of these from variation not explained by the other levels of the model by utilising a subject level. Finally, mPCA allows us to assess covariates that may have a subtle influence on facial shape, as the variation due to these covariates is less likely to be “hidden” during the dimension reduction process. An explanation of the relationship between mPCA [10,11,12] and multilevel methods implemented, e.g., using structural equation models (SEMs), is given by Timmerman [10]: “traditionally, SEM aims at inference, whereas CA is used as a descriptive technique”. Indeed, we use mPCA here as a purely descriptive approach for our data set.

Maternal smoking and alcohol consumption are two covariates that may have a subtle influence on the facial shape of non-syndromic adolescents. Maternal smoking and alcohol consumption are potential teratogens (agents that are capable of disrupting normal development during pregnancy). Maternal smoking may influence facial shape by inducing hypoxic states in the foetus [13] or through epigenetics [14,15]. Maternal smoking has been linked to craniosynostosis [16,17], head circumference [18,19,20], facial asymmetry due to hemifacial microsomia [21] and cleft lip and palate [16,22]. Maternal alcohol consumption during pregnancy has been linked to foetal alcohol syndrome (FAS). Common facial features associated with FAS include short palpebral fissures, reduced upper lip height, maxillary hypoplasia and a short nose [23], alongside cognitive difficulties [24]. Historically, FAS has been attributed to high levels of alcohol consumption during pregnancy. However, it could be hypothesised that the features affected in FAS may be influenced to a lesser extent when lower alcohol levels are consumed by mothers during pregnancy. 

It is clearly advantageous that expectant mothers reduce the exposure of their child to teratogens. Unfortunately, despite these risks, expectant mothers still smoke and consume alcohol during pregnancy. With the advent of social media, facial appearance is becoming an increasing priority to the general public. Therefore, demonstrating the effects of smoking and alcohol on normal facial development, to supplement the risk of syndrome development, may act as a further deterrent for expectant mothers. 

Previous studies have suggested that even low levels of alcohol and smoking during pregnancy may have an influence on the facial shape of one-year-old children [25]. However, others suggest that maternal alcohol consumption has a minimal effect on the facial shape of non-syndromic children at 15 years old [26]. Given that maternal smoking and alcohol consumption may have a subtle influence on the facial shape of adolescents, they provide excellent examples for demonstrating where mPCA can be useful. 

The aim of this study is therefore to investigate the influence of maternal smoking and alcohol consumption on the facial shape of their offspring at 15 years old. We demonstrate the advantages of using mPCA by using an extended mPCA model of four levels to assess the influence of (1) maternal smoking, (2) maternal alcohol consumption, (3) biological sex and (4) subject on the facial shape of non-syndromic English adolescents who presented without any obvious craniofacial defects. We also aim to demonstrate the advantages of mPCA, in particular the descriptive visualisations possible with mPCA. We provide the equivalent visualisations for conventional PCA as a comparison. It is hoped readers could extrapolate the potential advantages to their field of study. 

Maternal smoking and alcohol consumption were categorised according to exposure of the foetus during the 1st and/or 2nd trimesters of pregnancy. The effect of maternal smoking and maternal alcohol consumption (average 1–2 glasses per week) was minimal, with 0.66% and 0.48% of the variation in the 21 landmarks of non-syndromic offspring explained, respectively. 

## 2. Materials and Methods

This is a cross-sectional study, utilising the data available from the Avon Longitudinal Study of Parents and Children (ALSPAC). The ALSPAC Ethics and Law Committee, and the Local Research Ethics Committees, provided approval for the study (B3166). Informed consent for the use of data collected via questionnaires and clinics was obtained from participants following the recommendations of the ALSPAC Ethics and Law Committee at the time. 

The ALSPAC cohort includes the children and partners of women who were expected to give birth between 1 April 1991 and 31 December 1992. Further cohorts of children, who were missed from the original subject recruitment phase were subsequently recruited, with a total of 15,589 foetuses included. Of these, 14,901 were alive at 1 years old, with 3D facial scans available for 4747 children at 15 years old. The mean age of the subjects was 15.43 (+/− 0.28 SD) years old. Maternal smoking during the 1st and 2nd trimesters was determined via questionnaire at 18 weeks gestation. Maternal alcohol consumption during the 1st trimester and when the baby was first felt to have moved (as a proxy for the 2nd trimester) was also determined via questionnaire. These were added to the statistical model as separate levels. Biological sex was added as a confounder as a separate level in the model. The ALSPAC website contains details of all the data that is available through a fully searchable data dictionary and variable search tool (http://www.bristol.ac.uk/alspac/researchers/our-data/). Further details on the ALSPAC can be found at Boyd et al. [27] and Fraser et al. [28]. 

The 3D laser facial scans were collected in natural head position using Konica Minolta VI-900 laser scanners. Those with craniofacial abnormalities and twins/triplets were excluded from this study. The methodology for the laser scans can be found in Kau et al. [29]. To facilitate landmarking, the faces were standardised by aligning on a vertical cylinder and using mid-endocanthion as the origin [30]. The landmark configurations were all superimposed using generalised Procrustes analysis. The scaling of the faces attempts to isolate differences in shape. Further information on the image processing procedure can be found at Toma et al. [31]. 

**Figure 1:**
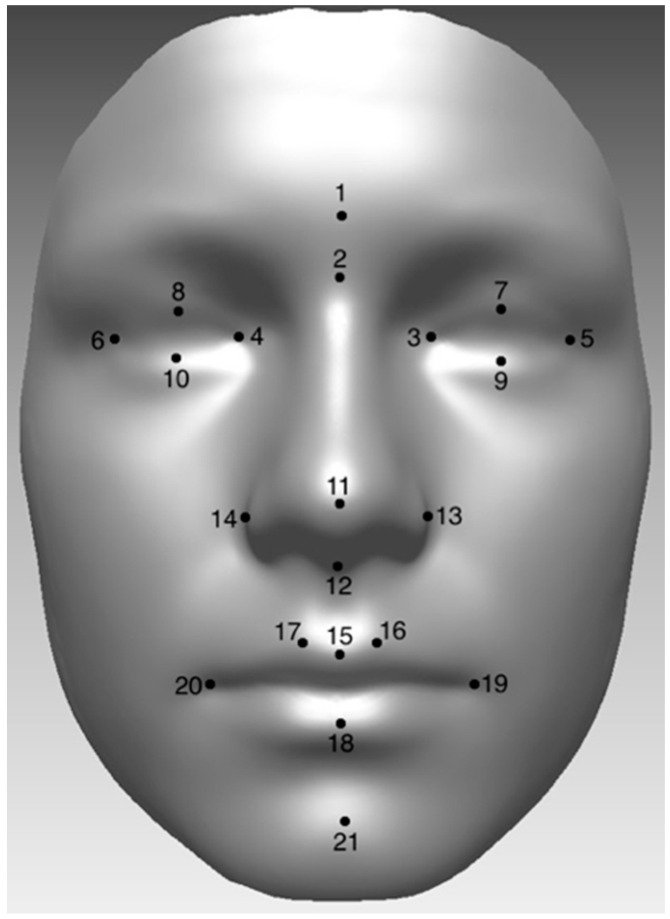
The 21 landmarks as described by Farkas [32]: (1) Glabella, (2) Nasion, (3) Endocanthion (Left), (4) Endocanthion (Right), (5) Exocanthion (Left), (6) Exocanthion (Right), (7) Palpebrale Superius (Left), (8) Palpebrale Superius (Right), (9) Palpebrale Inferius (Left), (10) Palpebrale Inferius (Right), (11) Pronasale, (12) Subnasale, (13) Alare (Left), (14) Alare (Right), (15) Labiale Superius, (16) Crista Phitri (Left), (17) Crista Philtri (Right), (18) Labiale Inferius, (19) Cheilion (Left), (20) Cheilion (Right), and (21) Pogonion.

The outcome measure used was differences in 21 facial landmarks (used as a proxy for the main facial features), as described by Farkas [32]. These were plotted manually onto 3D facial scans of the subjects, using Rapidform 2006 (Figure 1). The researcher plotting the landmarks underwent a calibration exercise and was blinded to the smoking and alcohol consumption of the subjects’ mothers during pregnancy. The facial images were plotted in grey scale to remove any influence of skin tone or makeup. The landmarks chosen are accurate and reproducible [33].

**Figure 2:**
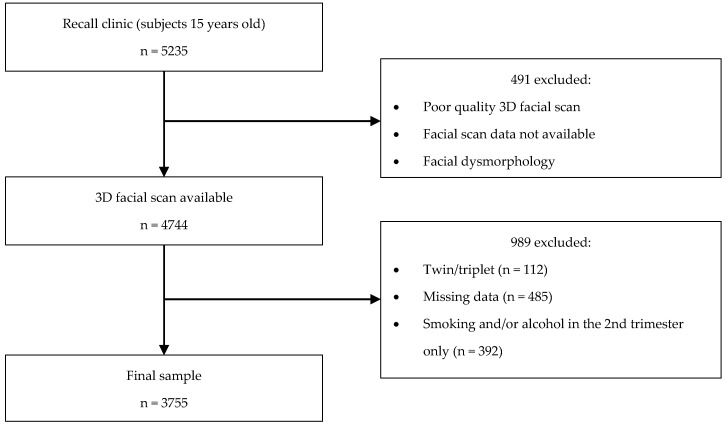
A flow chart of participant inclusion.

mPCA was used for the analysis. This method requires categorisation of the data. The sample sizes per grouping are presented in Table 1 and the flow of inclusion of subjects in Figure 2. A sample size calculation was not possible due to the novelty of the analysis. The mPCA technique does not currently manage missing data. Analyses were restricted in the following ways:

- It was not possible to assess the influence of smoking and alcohol levels throughout pregnancy (e.g., low/high) due to sample sizes in each grouping, and this is the subject of further work.
- Subjects who smoked or consumed alcohol in the 2nd trimester only were excluded due to reduced sample sizes in some of the groupings.

mPCA was used as a supervised dimension reduction technique and was carried out in MatLab 2017b. Maternal smoking, maternal alcohol consumption and biological sex of subject were explicitly modelled here. An attempt is made to control for “variation not explained by levels 1, 2 or 3” by adding a “subject” level to the model. For each of the following four levels, the eigenvalues and eigenvectors were found by determining covariance matrices for each level individually:

- Level one: Maternal smoking during pregnancy;
- Level two: Maternal alcohol consumption during pregnancy;
- Level three: Biological sex of the subject;
- Level four: Variations that are consistent across levels 1 to 3, i.e., the variation not explained by levels 1 to 3.

**Figure 3:**
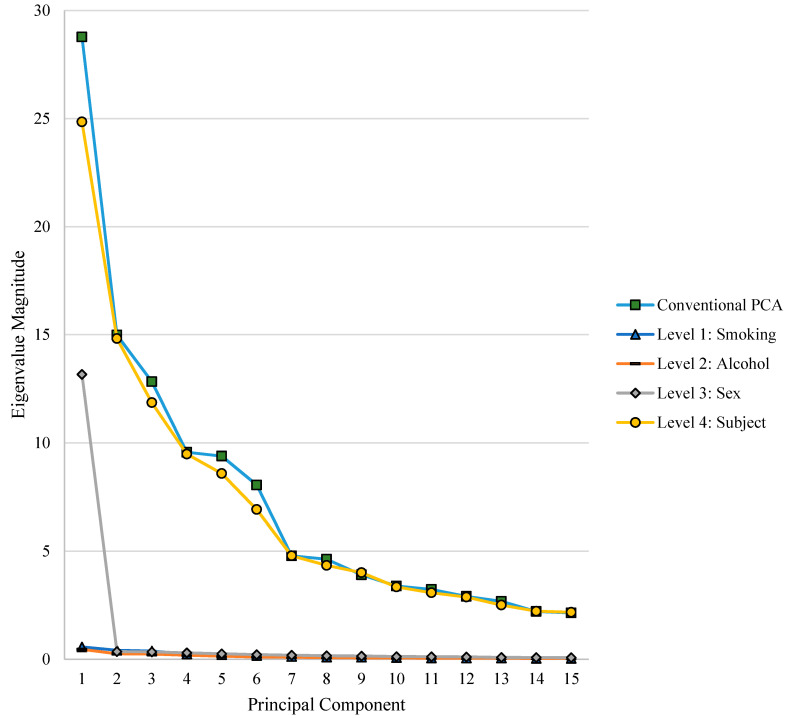
Eigenvalue plot allowing visualisation of the eigenvalue magnitude for each principal component, at each level of the model.

The number of eigenvalues retained in the mPCA analysis was determined by visualisation of the eigenvalue plot (Figure 3). The maximum number of eigenvalues that can be retained at each level is the number of groups minus 1, which is because the ranks of covariance matrices are constrained by the number of groups or subjects used in obtaining them. As sex only has two possible groupings (male/female), only one eigenvalue was retained at this level. 

The mPCA model is fitted to the new set of landmark points by using all four levels in the model at the same time. This is written as: new shape = grand mean shape (over all subjects, all smoking groups, all alcohol groups and both sexes) + weighted modes/components due to level 1 (maternal smoking) + weighted modes/components due to level 2 (maternal alcohol) + weighted modes/components due to level 3 (biological sex of subject) + weighted modes/components due to level 4 (subjective variations that are consistent across all groups). These weights are also called component "scores". The model fit was found by using a global optimisation procedure of an overall point-to-point cost function using MatLab. 

Farnell et al. [5,6,7,8,9] describe the mathematical concepts and various applications of mPCA, including nested, non-nested and mixed approaches. A non-nested approach was used here, with median averaging of the covariance matrices used to attempt to deal with outliers. The terms “nested” and “non-nested” refer to the population of subjects, shapes or images being studied, and the analysis follows the structure of the data. Fully “nested” cases are those where shapes, subjects, or groups belong to exactly and only one group in the level above it and (importantly) at all levels. A classic example here are clusters by school or class for some arbitrary “outcome” (e.g., exam results or here, facial shape). Each child belongs to one class only and each class belongs to only one school. Thus, this design is “fully nested” at all levels. Fully “non-nested” cases have groups at a given level that can belong to more than one group in the levels above it. Our dataset is a good example of a non-nested case. We represent maternal smoking, maternal alcohol consumption and biological sex at different levels (e.g., 1, 2 and 3) of the model and subjects at the bottom level (4). Note that one can have males and females in any of the smoking or alcohol groups. Note that there is no actual order as such for the smoking, alcohol and biological sex levels (1, 2 and 3) in this example, although we might impose an order on the data artificially. In practice, all that this would mean for our analysis using mPCA is that covariance matrices are found in a slightly different manner. Finally, some multilevel cases contain both fully nested and non-nested elements, which one might refer to as “mixed”. “Mixed” approaches are appropriate, for example, when assessing multiple images from the same subject. The use of mPCA in facial shape research can be seen as a complimentary descriptive technique to traditional statistical methods in this field. 

Estimates of the (standard) errors associated with the eigenvalues were found using Monte Carlo simulation (number of MC replicates = 1000). A simple Monte Carlo method was used here only in order to estimate the (standard) errors of eigenvalues. The mean shape vector (mu) and the covariance matrix (sigma) were found for the original data set of sample size *n*. Note that the *x*-, *y*-, and *z*-components of the landmark points in the original data set were found to be normally distributed. The MATLAB command “mvnrnd(mu,sigma,n)” was used to create Monte Carlo replicates of the data set, assuming an underlying (correlated) multivariate normal distribution. The 1000 MC replicates of the data were thus created, and eigenvalues were determined for each replicate. In this manner, standard deviations for the eigenvalues over the 1000 MC replicates provided broad estimates of standard errors. A limitation of this simple approach is that some of the complexity of the original data might be lost; future studies will consider more complicated MC schemes that retain this complexity.

In summary, the following analysis was conducted:

- Non-nested, 4-level analysis;
- Levels: 1) smoking, 2) alcohol, 3) sex, and 4) subject;
- Eigenvalues retained: smoking: 2, alcohol: 2, sex: 1, and subject: 35.

Conventional PCA was performed using the PCA function in MatLab 2017b in order to generate component scores for a comparison with mPCA. The sign attributed to the scores is reversed in comparison to those generated using the inhouse algorithm associated with mPCA. However, the patterns visualised in the data are the same.

The total percentage of variation explained by each mPCA level was calculated by dividing the retained eigenvalue magnitude for the respective levels by the total eigenvalue magnitude. The component scores for conventional PCA and each level of mPCA were standardised by dividing by the square root of the respective eigenvalue. Where appropriate, the differences in each of the 21 landmarks were determined by finding the difference between the landmarks of the average face plus 2*square root of the eigenvalue magnitude and average face minus 2*square root of the eigenvalue magnitude. 

## 3. Results

### 3.1. Demographics

In total, 1775 males and 1980 females were included in this study. Smoking and alcohol consumption levels of their mothers during pregnancy are presented in Table 1, with more detailed information available as supplementary material (Supplementary Tables S2–S5). Smoking during pregnancy was rarer than alcohol consumption. In total, 593 (15.8%) mothers smoked during the 1st trimester, with 159 of the mothers included in the study stopping smoking before the 2nd trimester. The smoking levels of mothers in the 1st and 2nd trimester varied, with a mean ranging from 5 to 11 cigarettes per day in each group. In total, 2378 mothers (63.3%) consumed alcohol within the 1st trimester of pregnancy and 1798 mothers (47.9%) during the 1st and 2nd trimesters. Alcohol consumption during pregnancy was therefore relatively common for the ALSPAC mothers in 1990. The alcohol consumption levels of mothers in the 1st and 2nd trimesters varied from <1 glass to 2 glasses per week on average. Very high levels of alcohol consumption were rare, with five mothers reporting drinking 3–9 glasses/day and two mothers reporting 10+ glasses/day (Supplementary Tables S4 and S5). The mothers were asked to take “glass” to mean “a pub measure of spirits, half a pint of lager/cider, a glass of wine etc.”.

### 3.2. Maternal Smoking Level

One of the main benefits of mPCA is that we can easily calculate the percentage of the total variation due to maternal smoking. Analysis of the eigenvalues reveals that the maternal smoking level had a minimal influence (0.66%) on the total variation in the 21 facial landmarks (Table 2, Figure 3). Estimates of the (standard) errors associated with the eigenvalues found using Monte Carlo simulation are shown in Supplementary Table S1. Given that a small amount of variation is attributed to maternal smoking, it follows that the variance associated with this covariate is small. Therefore, in conventional PCA, the true influence of maternal smoking may be “hidden” in multiple PCs, particularly if a large amount of the variance associated with these PCs is explained by another covariate.

**Figure 4:**
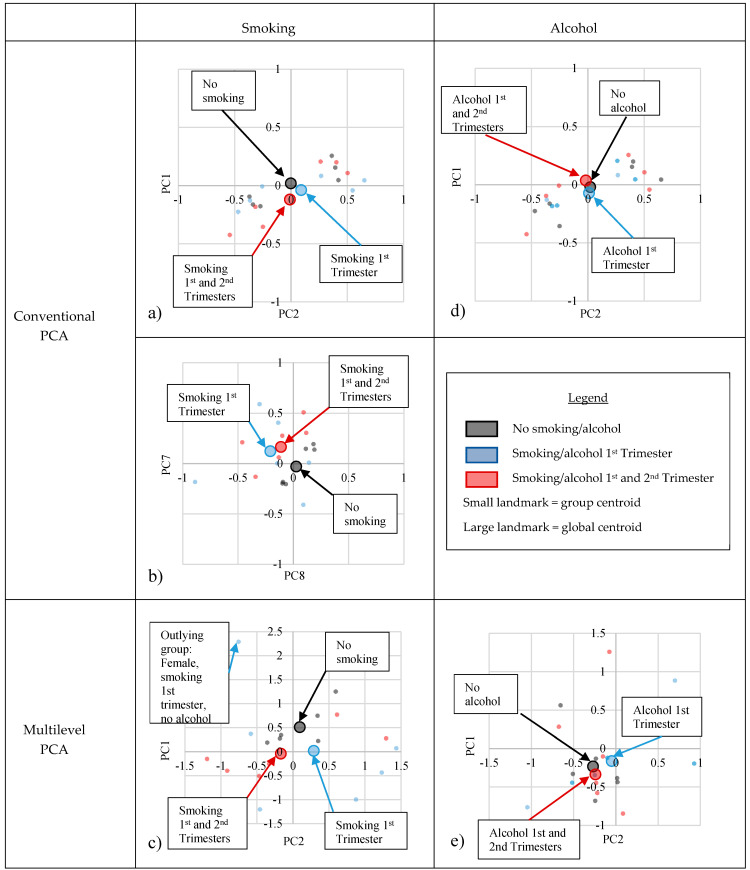
Scatter plots of the standardised component scores at PC1 and PC2 for conventional PCA (**a**,**b**,**d**) and mPCA (**c**,**e**). No obvious pattern in the separation of the group means is evident in conventional PCA PC1/2 (**a**). There is a suggestion of a pattern in conventional PCA PC7/8 (**b**) and mPCA smoking level PC2 (**c**). Subjects whose mothers did not smoke during pregnancy are possibly separated from those whose mothers smoked during the 1st trimester or both the 1st and 2nd trimesters. There is no obvious pattern in the separation of the group means with alcohol consumption during pregnancy (**d**,**e**). Scatter plots for conventional PCA PC3–10 are available as supplementary material.

The standardised component scores for conventional PCA PC1 and PC2 do not show any obvious patterns in the separation of the group means (Figure 4a). Inspection of the component scores up to PC10 suggests that there may be some subtle separation of the group means due to maternal smoking at PC7/8 (Figure 4b, Supplementary Figure S1). However, as discussed previously, when using conventional PCA, it is difficult to assess this with certainty from a visual plot, as each PC cannot be solely attributed to one covariate. This is because the PCs are orthogonal and were generated in an unsupervised manner, not taking into account any of the covariates at the beginning of the analysis. The variation explained by PC7/8 may include the influence of maternal smoking but will be “mixed in” with variation due to other covariates. 

Multilevel PCA allows us to be more certain about the differences we visualise, as the PCs at level one were generated taking maternal smoking into account. Indeed, we see a similar pattern in the separation of the group means at PC2 of the mPCA model as we see with conventional PCA PC7/8. The offspring of mothers who smoked in the 1st trimester only and smoked in both the 1st and 2nd trimesters are possibly differentiable from those whose mothers did not smoke during pregnancy (Figure 4c). The ease at which any potential patterns can be visualised demonstrates one of the strengths of mPCA. Eight PCs needed to be visualised when using conventional PCA to gain a suggestion of the influence of maternal smoking, whereas only two PCs needed to be visualised with mPCA. This is more efficient. 

Our analyses suggest that the nasal bridge shape/length and orbital position/shape may be most influenced by the maternal smoking level. Differences were also seen in the prominence of the brow-ridge, lower lip and chin, and the position of the corners of the mouth (Table 3). These differences may suggest that smoking could influence shared facial genes. However, as there is an outlying group of females who smoked in the 1st trimester and consumed no alcohol (Figure 4c), only a suggestion of a pattern in the separation of the group means, differential sample sizes in the groups and a large range of component scores in each grouping (Table 4), any differences should be interpreted with caution at this stage.

### 3.3. Maternal Alcohol Consumption Level

The maternal alcohol consumption level, in this population, influenced the variation in the 21 facial landmarks by 0.48%, and thus had a minimal effect (Table 2, Figure 3). Estimates of the (standard) errors associated with the eigenvalues found using Monte Carlo simulation are shown in Supplementary Table S1. The scatter plots of the population centroids suggest that there is little pattern in the separation of the group means at this level in mPCA (Figure 4d) and when visualising the first 10 PCs of conventional PCA (Figure 4c, Supplementary Figure S1). This further confirms that alcohol consumption in this population does not appear to have had a long-term influence on the children’s main facial features. However, as the maternal alcohol consumption in this population was generally low at 1–2 glasses per week, and only non-syndromic children without obvious craniofacial abnormality were assessed here, this finding does not condone the consumption of alcohol during pregnancy.

### 3.4. Biological Sex Level 

**Figure 5:**
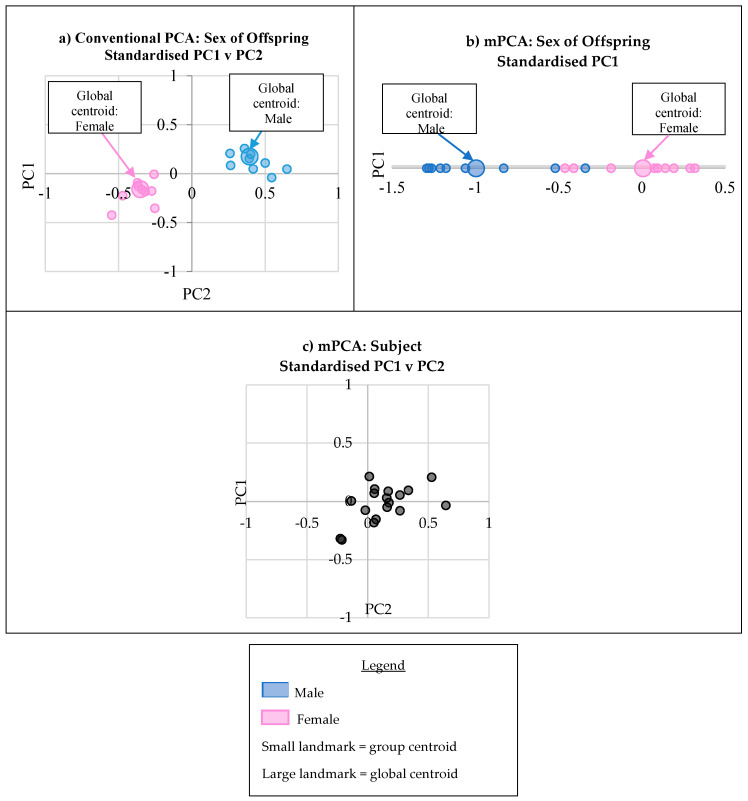
Scatter plots of the standardised component scores at PC1 and PC2 for conventional PCA (**a**), PC1 for sex level mPCA (**b**) and PC1 and PC2 for subject level mPCA (**c**). Clear separation of the group means of the biological sexes is evident for both conventional PCA and mPCA. At a subject level, the centroids seem to be centred around the origin, although there is some deviation from this, perhaps due to group sample sizes.

Multilevel PCA suggests that the biological sex level explains 8.88% of the variation in the 21 facial landmarks (Table 2, Figure 3). Estimates of the (standard) errors associated with the eigenvalues found using Monte Carlo simulation are shown in Supplementary Table S1. Conventional PCA shows that there is a clear pattern in the separation of the group means of the subjects due to biological sex when visualising the component scores at PC1 and PC2 (Figure 5a). However, as discussed previously, it is very likely that other covariates are contributing to this effect. The effects of the unknown covariates may enhance or reduce the separation that we see. Clear separation of the group means by biological sex is also visible at mPCA PC1 (Figure 5b). Only one PC is possible in mPCA due to the rank of the matrices. 

### 3.5. Subject Variation (Variation not Explained by Levels 1–3)

Subject variation is not explicitly modelled in conventional PCA. Instead the effects of other covariates are “mixed in” with the effects of maternal smoking, maternal alcohol consumption and biological sex. Being able to separate these effects in mPCA is a distinct advantage. Multilevel PCA suggests that 85.85% of the variation in the 21 landmarks is not due to smoking, alcohol or biological sex (Table 2). Estimates of the (standard) errors associated with the eigenvalues found using Monte Carlo simulation are shown in Supplementary Table S1. It follows that in conventional PCA, the effect of other covariates (not smoking, alcohol or biological sex) on the PCs, and thus the component scores, is high. This can be visualised in the eigenvalue plot, where the eigenvalues for conventional PCA follow a similar pattern as the subject level eigenvalues in mPCA (Figure 3).

When visualising the standardised component scores from the subject level of mPCA, the population centroids by any of the groupings (i.e., maternal smoking, maternal alcohol level or biological sex) are generally centred at the origin at the subject level (Figure 5c), as anticipated. There are a few groups that are more distant to the origin, which may be explained by the small sample sizes in some of the groups.

## 4. Discussion

### 4.1. Smoking

The influence of maternal smoking during pregnancy on non-syndromic facial shape appears to be subtle (0.66% of the total variation in the 21 landmarks). The differences in the facial features in this population appear to be most evident when comparing subjects whose mothers did not smoke in pregnancy with those whose mothers smoked in the 1st trimester or the 1st and 2nd trimesters. A previous study on a one-year-old population suggested in their supplementary material that some minor changes, in the region of 0.4–0.5 mm, may be seen in the chin, lower lip prominence, forehead prominence and nasal bridge shape [25]. Our findings agree with most of these findings, with the addition of possible differences in the orbits. However, given that the differences explain less than 1% of the total variation in the 21 landmarks, our overall findings also agree with Koziel et al. [34], who suggested that, after 10 years old, the influence of maternal smoking may be minimal, particularly in females. It should also be noted that our findings are based on individuals who have no history of craniofacial abnormality, do not separate high/low levels of exposure and assessed 21 facial landmarks only. This study primarily aims to provide an example of mPCA being used for the exploration of environment factors on facial shape at this stage and thus a more detailed analysis is the subject of further work.

### 4.2. Alcohol

There have been conflicting results on the impact of low levels of maternal alcohol consumption on facial shape in the literature. Muggli et al. [25] suggested that alcohol during the 1st trimester could have an impact on facial shape at 1 years old. They suggest that low levels of alcohol consumption could influence forehead shape, moderate to high alcohol consumption could affect orbital shape, midface and chin prominence, and binge levels of alcohol could affect the chin. These differences appear to be in the range of 1 mm. Conversely, Howe et al. [26], who also looked at the influence of low alcohol consumption on the ALSPAC cohort, found that alcohol had minimal effect on facial shape at 15 years old, using alternative methodology to mPCA. It could therefore be hypothesised that any change in facial shape due to maternal alcohol (without a FAS diagnosis) is overshadowed by the influence of other factors as the child develops into adolescence. However, these differences may also be explained by the fact that different populations and different methodologies were used.

### 4.3. Strengths and Limitations of mPCA

This study is a starting point, using mPCA methodology in the field of facial shape research. mPCA methodology is a flexible dimension reduction technique. Separating the covariates into levels allows us to be more confident that subtle variation due to smoking and alcohol exposure during pregnancy was retained and increases confidence that the differences visualised between the groups using scatter plots were due to the covariate assessed. The addition of a “subject” level has also attempted to isolate the effect of variation not explained by smoking, alcohol or sex. As many of the covariates in facial shape research are likely to be unknown, studies which have utilised alternative methodologies may not correct for all of the necessary covariates. The inclusion of a “subject” level is therefore a step towards modelling this unknown variation. 

As mPCA requires categorisation of the covariates, it can be difficult to assess naturally continuous variables. Furthermore, as the number of eigenvalues retained is limited by the rank of the matrices, the number of eigenvalues available for assessment is reduced. The addition of inferential evidence is clearly the next step for this analysis and work is ongoing to develop a bootstrap approach to this. 

### 4.4. Strengths and Limitations of Study

This study utilises a large overall sample and begins to investigate the long-term impact of maternal smoking and alcohol consumption on the facial shape of offspring. Previous studies have assessed the influence of possible dose-dependent relationships and the influence of the individuals’ alcohol susceptibility [25]. It was not possible to assess these factors in this population, using this methodology, due to sample size restrictions. This, however, could be the subject of further work. Further, smoking and alcohol exposures were determined via self-questionnaire, which have conflicting levels of reliability in the literature [35,36]. Furthermore, the image data was reduced to 21 facial landmarks in this study. Further work will focus on the use of many more landmarks through the form of dense facial meshes, such as those used in Muggli et al. [25]. Scaled faces were used here in order to isolate the influence on facial shape, as is common in facial shape research, but this removes the influence on size. Investigating the influence on facial shape and size may be interesting in future. Finally, the influence of protective factors such as folic acid [37] and multivitamins [38] alongside the influence of nicotine replacement therapy, paternal smoking during pregnancy, and smoking exposure after pregnancy would all also be interesting to investigate in future.

## 5. Conclusions

- mPCA is a useful descriptive tool for assessing the influence of environmental factors on facial shape.
- The effect of maternal smoking and maternal alcohol consumption (average 1–2 glasses per week) was minimal, with 0.66% and 0.48% of the variation in the 21 landmarks of non-syndromic offspring explained, respectively.

## Figures and Tables

**Table 1:** Subject demographics ^1^.

Grouping	Sex	Sample Size	Age	Smoking Levels Mean +/- SD (Cigarettes/Day)		Alcohol Levels Mean +/- SD (Glasses/Week) ^2^	
				1st Trimester	2nd Trimester	1st Trimester	2nd Trimester
No smoking No alcohol	Male	574	15.41 +/- 0.25	-	-	-	-
	Female	622	15.42 +/- 0.28	-	-	-	-
No smoking Alcohol 1st trimester only	Male	214	15.42 +/- 0.26	-	-	0.82 +/- 2.91	-
	Female	275	15.40 +/- 0.25	-	-	0.68 +/- 0.90	-
No smoking Alcohol 1st and 2nd trimesters	Male	702	15.42 +/- 0.27	-	-	1.17 +/- 3.40	1.22 +/- 3.42
	Female	775	15.44 +/- 0.30	-	-	1.02 +/- 2.04	1.10 +/- 2.87
Smoking 1st trimester only No alcohol	Male	23	15.51 +/- 0.30	7.10 +/- 7.16	-	-	-
	Female	18	15.60 +/- 0.34	7.19 +/- 5.35	-	-	-
Smoking 1st trimester only Alcohol 1st trimester only	Male	15	15.33 +/- 0.14	5.50 +/- 4.27	-	2.07 +/- 3.46	-
	Female	18	15.56 +/- 0.45	9.26 +/- 6.28	-	1.24 +/- 2.22	-
Smoking 1st trimester only Alcohol 1st and 2nd trimester	Male	41	15.40 +/- 0.25	7.24 +/- 6.30	-	1.16 +/- 1.56	0.84 +/- 0.47
	Female	44	15.53 +/- 0.42	7.77 +/- 6.74	-	2.26 +/- 6.40	1.25 +/- 2.07
Smoking 1st and 2nd trimesters No alcohol	Male	68	15.44 +/- 0.28	10.48 +/- 6.27	10.47 +/- 7.40	-	-
	Female	72	15.47 +/- 0.36	10.88 +/- 6.90	11.47 +/- 6.34	-	-
Smoking 1st and 2nd trimesters Alcohol 1st trimester only	Male	23	15.41 +/- 0.28	10.70 +/- 7.06	8.48 +/- 5.94	0.67 +/- 0.38	-
	Female	35	15.44 +/- 0.32	11.19 +/- 5.73	9.56 +/- 6.33	0.70 +/- 0.40	-
Smoking 1st and 2nd trimesters Alcohol 1st and 2nd trimesters	Male	115	15.40 +/- 0.21	10.54 +/- 6.81	9.66 +/- 6.56	1.97 +/- 5.63	1.20 +/- 1.83
	Female	121	15.42 +/- 0.25	11.26 +/- 7.64	10.81 +/- 7.46	2.29 +/- 5.81	1.28 +/- 2.15

^1^ The raw data received from the Avon Longitudinal Study of Parents and Children (ALSPAC) had been previously categorised. The sample means were therefore calculated using: mean = sum(frequency x mid interval point of category)/sum frequency. The frequency tables are available as supplementary material (Supplementary Tables S2–S5). ^2^ The mothers were asked to take “glass” to mean “a pub measure of spirits, half a pint of lager or cider, a glass of wine etc.”.

**Table 2:** Percentage of the total variation in the 21 facial landmarks as explained by each level.

Level	% of Total Variation in 21 Landmarks		
	PC1	PC2	All Retained PCs
Level 1: Maternal Smoking	0.38%	0.28%	0.66%
Level 2: Maternal Alcohol Consumption	0.31%	0.17%	0.48%
Level 3: Biological Sex	8.88%	N/A	8.88%
Level 4: Subject	16.75%	9.99%	85.85%

**Table 3:** Differences in each landmark due to maternal smoking level PC2. These results should be interpreted with caution at this stage. They should be taken as a suggestion of which landmarks are likely to vary due to maternal smoking rather than providing exact values.

Landmark	Difference in mm between Average Face + 2* Square Root Eigenvalue and Average Face − 2* Square Root Eigenvalue		
	x	y	z
Glabella	−0.08	−0.24	−0.53
Nasion	−0.02	−0.84	−0.22
Endocanthion (Left)	−0.76	−0.11	0.20
Endocanthion (Right)	0.88	0.04	0.05
Exocanthion (Left)	0.41	0.35	0.27
Exocanthion (Right)	−0.07	0.32	0.02
Palpebrale Superius (Left)	−0.51	0.45	−0.15
Palpebrale Superius (Right)	0.50	0.30	−0.23
Palpebrale Inferius (Left)	−0.47	0.15	−0.09
Palpebrale Inferius (Right)	0.54	0.11	0.04
Pronasale	−0.11	0.86	0.70
Subnasale	−0.14	0.60	0.08
Alare (Left)	−0.49	0.07	0.18
Alare (Right)	0.29	0.20	0.12
Labiale Superius	−0.07	−0.30	−0.24
Labiale Inferius	−0.07	−0.20	0.56
Crista Phitri (Left)	−0.13	−0.42	−0.27
Crista Phitri (Right)	−0.07	−0.33	−0.20
Cheilion (Left)	0.25	−0.62	−0.65
Cheilion (Right)	−0.04	−0.65	−0.37
Pogonion	0.18	0.25	0.50

**Table 4:** Mean standardised component score and standard deviation for each grouping.

Grouping	Sex	Smoking Level mPCA				Alcohol Level mPCA			
		PC1	SD	PC2	SD	PC1	SD	PC2	SD
No smoking No alcohol	Male	−0.359	5.997	0.195	5.104	−0.238	3.302	−0.128	3.720
	Female	−0.184	5.918	0.277	4.734	0.012	3.360	−0.382	3.381
No smoking Alcohol 1st trimester only	Male	0.591	5.701	1.255	4.758	−0.252	3.365	−0.342	3.606
	Female	0.339	6.222	0.752	4.324	−0.101	3.658	−0.117	3.607
No smoking Alcohol 1st and 2nd trimesters	Male	0.346	5.852	0.227	4.968	−0.156	3.605	−0.099	3.709
	Female	−0.167	5.856	0.348	4.894	−0.236	3.689	−0.444	3.396
Smoking 1st trimester only No alcohol	Male	1.433	4.691	0.076	3.510	0.016	2.796	−0.433	3.262
	Female	−0.758	5.298	2.291	4.108	−0.656	2.897	0.565	3.842
Smoking 1st trimester only Alcohol 1st trimester only	Male	0.873	4.711	−0.992	3.265	0.697	2.742	0.887	4.030
	Female	−0.461	6.934	−1.199	3.381	−1.051	3.456	−0.762	3.747
Smoking 1st trimester only Alcohol 1st and 2nd trimester	Male	−0.587	6.301	0.375	5.091	−0.677	2.940	0.285	3.411
	Female	1.231	5.813	−0.431	5.318	−0.079	3.111	1.261	2.944
Smoking 1st and 2nd trimesters No alcohol	Male	−0.332	4.674	−0.249	4.717	−0.247	3.411	−0.678	3.841
	Female	1.291	6.394	0.283	4.985	−0.511	3.141	−0.326	2.925
Smoking 1st and 2nd trimesters Alcohol 1st trimester only	Male	−0.472	6.068	−0.501	5.569	0.925	4.009	−0.192	3.597
	Female	−0.915	5.861	−0.392	5.124	−0.519	3.733	−0.441	3.825
Smoking 1st and 2nd trimesters Alcohol 1st and 2nd trimesters	Male	0.609	5.788	0.774	4.641	−0.223	3.489	−0.575	3.266
	Female	−1.195	5.121	−0.146	4.663	0.082	3.534	−0.843	3.265

## Supplementary Materials

The following are available online at https://www.mdpi.com/2313-433X/6/5/34/s1, Figure S1: Scatter plots of the standardized component scores for conventional PCA up to PC10. Group centroids (18 groups) are represented as smaller landmarks, and global centroids are represented as larger landmarks. A subtle pattern in the separation of the group means is visible at PC7/8, with the global centroid of individuals whose mothers did not smoke during pregnancy distant to the global centroid of those whose mothers smoked during pregnancy. No other obvious patterns in the separation of the group means is visualised. Table S1: First 10 eigenvalues for conventional PCA and mPCA (four levels). Estimates of the (standard) errors (SEs) associated with the eigenvalues can be found using Monte Carlo simulation (number of MC replicates = 1000), assuming a multivariate normal distribution for the landmark point data. Table S2: Frequencies and mean smoking levels (smoking, 1st trimester); Table S3: Frequencies and mean smoking levels (smoking, 2nd trimester); Table S4: Frequencies and mean alcohol levels (alcohol, 1st trimester); Table S5: Frequencies and mean alcohol levels (alcohol, 2nd trimester).
