# Supplementary material for: Multilevel Analysis of the Influence of Maternal Smoking and Alcohol Consumption on the Facial Shape of English Adolescents
Source: J Imaging. 2020 May 18;6(5):34. doi: 10.3390/jimaging6050034 (PMC8321032; doi:10.3390/jimaging6050034)
Supplement: Supplementary file 1 [file jimaging-06-00034-s001.pdf]

Table S1. First 10 eigenvalues for conventional PCA and mPCA (four levels). Estimates of the (standard) errors (SEs) associated with the eigenvalues can be found using Monte Carlo simulation (number of MC replicates = 1000), assuming a multivariate normal distribution for the landmark point data.

Eigenvalue Number	SL PCA	SD	mPCA Level 1 (Smoking)	SD	mPCA Level 2 (Alcohol)	SD	mPCA Level 3 (Sex)	SD	mPCA Level 4 (Subject)	SD
1	28.777	0.665	0.567	0.199	0.453	0.161	13.166	0.105	24.851	0.871
2	14.983	0.326	0.415	0.085	0.249	0.067	0.349	0.041	14.823	0.410
3	12.829	0.288	0.374	0.054	0.235	0.043	0.335	0.026	11.862	0.387
4	9.564	0.185	0.243	0.036	0.184	0.028	0.293	0.019	9.482	0.249
5	9.386	0.185	0.209	0.025	0.135	0.021	0.248	0.014	8.581	0.240
6	8.048	0.186	0.169	0.019	0.103	0.016	0.210	0.011	6.915	0.254
7	4.781	0.096	0.123	0.014	0.090	0.013	0.184	0.009	4.785	0.131
8	4.627	0.095	0.099	0.012	0.072	0.010	0.160	0.007	4.342	0.123
9	3.902	0.089	0.094	0.009	0.057	0.008	0.146	0.006	4.004	0.131
10	3.387	0.074	0.078	0.008	0.053	0.007	0.121	0.005	3.343	0.095

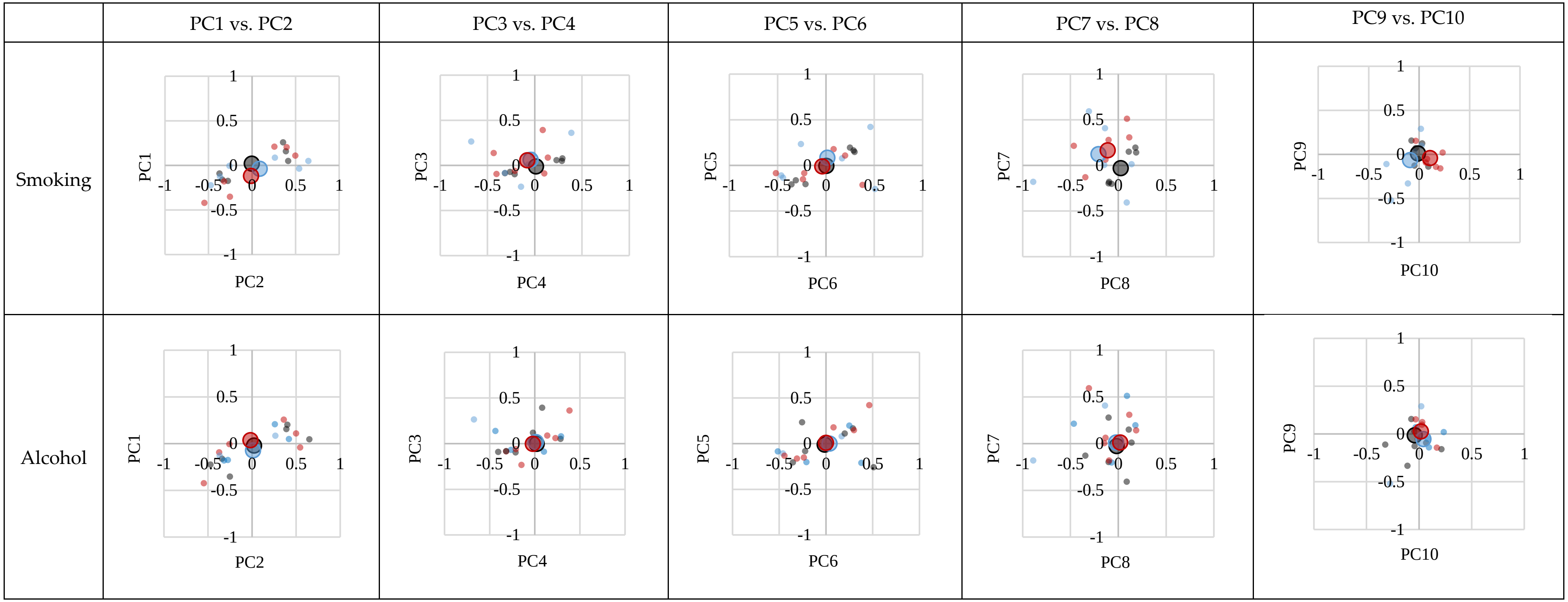

Figure S1: Scatter plots of the standardized component scores for conventional PCA up to PC10. Group centroids (18 groups) are represented as smaller landmarks, and global centroids are represented as larger landmarks. A subtle pattern in the separation of the group means is visible at PC7/8, with the global centroid of individuals whose mothers did not smoke during pregnancy distant to the global centroid of those whose mothers smoked during pregnancy. No other obvious patterns in the separation of the group means is visualised.

<u>Legend</u>

No smoking/alcohol

Smoking/alcohol 1st Trimester

Smoking/alcohol 1st and 2nd Trimester

Small landmark = group centroid

Large landmark = global centroid

Table S2. Frequencies and mean smoking levels (smoking, 1st trimester)

Grouping	Sex	Smoking Levels	Mid Interval Value (m)	Frequency (f)	Mean (cigarettes/day)	Standard Deviation (cigarettes/day)
Smoking 1st trimester only No alcohol	Male	1–4 cigs/day	2.5	14	7.10	7.16
		5–9 cigs/day	7	<5		
		10–14 cigs/day	12	<5		
		15–19 cigs/day	17	<5		
		20–24 cigs/day	22	<5		
		25–29 cigs/day	27.5	<5		
		30+ cigs/day	30	<5*		
	Female	1–4 cigs/day	<5	7	7.19	5.35
		5–9 cigs/day	7	7		
		10–14 cigs/day	12	<5		
		15–19 cigs/day	17	<5		
		20–24 cigs/day	22	<5		
		25–29 cigs/day	27.5	<5*		
		30+ cigs/day	30	<5*		
Smoking 1st trimester only Alcohol 1st trimester only	Male	1–4 cigs/day	2.5	8	5.5	4.27
		5–9 cigs/day	7	<5		
		10–14 cigs/day	12	<5		
		15–19 cigs/day	17	<5		
		20–24 cigs/day	22	<5*		
		25–29 cigs/day	27.5	<5*		
		30+ cigs/day	30	<5*		
	Female	1–4 cigs/day	2.5	5	9.26	6.28
		5–9 cigs/day	7	6		
		10–14 cigs/day	12	5		
		15–19 cigs/day	17	<5		
		20–24 cigs/day	22	<5*		
		25–29 cigs/day	27.5	<5		
		30+ cigs/day	30	<5*		
Smoking 1st trimester only Alcohol 1st and 2nd trimester	Male	1–4 cigs/day	2.5	19	7.24	6.30
		5–9 cigs/day	7	12		
		10–14 cigs/day	12	6		
		15–19 cigs/day	17	<5*		
		20–24 cigs/day	22	<5		
		25–29 cigs/day	27.5	<5		
		30+ cigs/day	30	<5*		
	Female	1–4 cigs/day	2.5	22	7.77	6.74
		5–9 cigs/day	7	7		
		10–14 cigs/day	12	8		
		15–19 cigs/day	17	<5		
		20–24 cigs/day	22	<5		
		25–29 cigs/day	27.5	<5*		
		30+ cigs/day	30	<5		
Smoking 1st and 2nd trimesters No alcohol	Male	1–4 cigs/day	2.5	16	10.48	6.27
		5–9 cigs/day	7	13		
		10–14 cigs/day	12	23		
		15–19 cigs/day	17	12		
		20–24 cigs/day	22	<5		
		25–29 cigs/day	27.5	<5		
		30+ cigs/day	30	<5		
	Female	1–4 cigs/day	2.5	17	10.88	6.90
		5–9 cigs/day	7	18		
		10–14 cigs/day	12	14		
		15–19 cigs/day	17	14		
		20–24 cigs/day	22	7		
		25–29 cigs/day	27.5	<5		
		30+ cigs/day	30	<5*		
Smoking 1st and 2nd trimesters Alcohol 1st trimester only	Male	1–4 cigs/day	2.5	<5	10.70	7.06
		5–9 cigs/day	7	9		
		10–14 cigs/day	12	<5		
		15–19 cigs/day	17	<5		
		20–24 cigs/day	22	<5		

		25–29 cigs/day	27.5	<5*		
		30+ cigs/day	30	<5		
	Female	1–4 cigs/day	2.5	<5	11.19	5.73
		5–9 cigs/day	7	14		
		10–14 cigs/day	12	8		
		15–19 cigs/day	17	6		
		20–24 cigs/day	22	<5		
		25–29 cigs/day	27.5	<5*		
		30+ cigs/day	30	<5*		
Smoking 1st and 2nd trimesters Alcohol 1st and 2nd trimesters	Male	1–4 cigs/day	2.5	31	10.54	6.81
		5–9 cigs/day	7	20		
		10–14 cigs/day	12	37		
		15–19 cigs/day	17	13		
		20–24 cigs/day	22	10		
		25–29 cigs/day	27.5	<5		
		30+ cigs/day	30	<5*		
	Female	1–4 cigs/day	2.5	28	11.26	7.64
		5–9 cigs/day	7	34		
		10–14 cigs/day	12	20		
		15–19 cigs/day	17	18		
		20–24 cigs/day	22	13		
		25–29 cigs/day	27.5	7		
		30+ cigs/day	30	<5		

*This many contain zero.

Table S3. Frequencies and mean smoking levels (smoking, 2nd trimester).

Grouping	Sex	Smoking Levels	Mid Interval Value (m)	Frequency (f)	Mean (cigarettes/day)	Standard Deviation (cigarettes/day)
Smoking 1st and 2nd trimesters No alcohol	Male	1–4 cigs/day	2.5	19	10.47	7.40
		5–9 cigs/day	7	17		
		10–14 cigs/day	12	13		
		15–19 cigs/day	17	10		
		20–24 cigs/day	22	6		
		25–29 cigs/day	27.5	<5		
		30+ cigs/day	30	<5		
	Female	1–4 cigs/day	2.5	13	11.47	6.34
		5–9 cigs/day	7	16		
		10–14 cigs/day	12	19		
		15–19 cigs/day	17	16		
		20–24 cigs/day	22	7		
		25–29 cigs/day	27.5	<5		
		30+ cigs/day	30	<5*		
Smoking 1st and 2nd trimesters Alcohol 1st trimester only	Male	1–4 cigs/day	2.5	8	8.48	5.94
		5–9 cigs/day	7	6		
		10–14 cigs/day	12	6		
		15–19 cigs/day	17	<5		
		20–24 cigs/day	22	<5		
		25–29 cigs/day	27.5	<5*		
		30+ cigs/day	30	<5*		
	Female	1–4 cigs/day	2.5	9	9.56	6.33
		5–9 cigs/day	7	12		
		10–14 cigs/day	12	6		
		15–19 cigs/day	17	<5		
		20–24 cigs/day	22	<5		
		25–29 cigs/day	27.5	<5*		
		30+ cigs/day	30	<5*		
Smoking 1st and 2nd trimesters Alcohol 1st and 2nd trimesters	Male	1–4 cigs/day	2.5	34	9.66	6.56
		5–9 cigs/day	7	28		
		10–14 cigs/day	12	28		
		15–19 cigs/day	17	14		
		20–24 cigs/day	22	9		
		25–29 cigs/day	27.5	<5		
		30+ cigs/day	30	<5		
	Female	1–4 cigs/day	2.5	31	10.81	7.46
		5–9 cigs/day	7	32		
		10–14 cigs/day	12	23		
		15–19 cigs/day	17	15		
		20–24 cigs/day	22	14		
		25–29 cigs/day	27.5	5		
		30+ cigs/day	30	<5		

*This many contain zero.

Table S4. Frequencies and mean alcohol levels (alcohol, 1st trimester)[1].

Grouping	Sex	Alcohol Levels[2]	Mid Interval Value [per week] (m)	Frequency (f)	Mean (glasses/week)	Standard Deviation (glasses/week)
No smoking Alcohol 1st trimester only	Male	<1 glass/week	0.5	195	0.82	2.91
		1–2 glasses/week	1.5	17		
		1–2 glasses/day	10.5	<5		
		3–9 glasses/day	42	<5		
		10+ glasses/day	70	<5*		
	Female	<1 glass/week	<5	243	0.68	0.90
		1–2 glasses/week	<5	30		
		1–2 glasses/day	10.5	<5		
		3–9 glasses/day	42	<5*		
		10+ glasses/day	70	<5*		
No smoking Alcohol 1st and 2nd trimesters	Male	<1 glass/week	0.5	491	1.17	3.40
		1–2 glasses/week	1.5	192		
		1–2 glasses/day	10.5	17		
		3–9 glasses/day	42	<5		
		10+ glasses/day	70	<5		
	Female	<1 glass/week	<5	550	1.02	2.04
		1–2 glasses/week	<5	209		
		1–2 glasses/day	10.5	15		
		3–9 glasses/day	42	<5		
		10+ glasses/day	70	<5*		
Smoking 1st trimester only Alcohol 1st trimester only	Male	<1 glass/week	0.5	10	2.07	3.46
		1–2 glasses/week	1.5	<5		
		1–2 glasses/day	10.5	<5		
		3–9 glasses/day	42	<5*		
		10+ glasses/day	70	<5*		
	Female	<1 glass/week	0.5	14	1.24	2.22
		1–2 glasses/week	1.5	<5		
		1–2 glasses/day	10.5	<5		
		3–9 glasses/day	42	<5*		
		10+ glasses/day	70	<5*		
Smoking 1st trimester only Alcohol 1st and 2nd trimester	Male	<1 glass/week	0.5	23	1.16	1.56
		1–2 glasses/week	1.5	17		
		1–2 glasses/day	10.5	<5		
		3–9 glasses/day	42	<5*		
		10+ glasses/day	70	<5*		
	Female	<1 glass/week	0.5	25	2.26	6.40
		1–2 glasses/week	1.5	16		
		1–2 glasses/day	10.5	<5		
		3–9 glasses/day	42	<5		
		10+ glasses/day	70	<5*		
Smoking 1st and 2nd trimesters Alcohol 1st trimester only	Male	<1 glass/week	0.5	19	0.67	0.38
		1–2 glasses/week	1.5	<5		
		1–2 glasses/day	10.5	<5*		
		3–9 glasses/day	42	<5*		
		10+ glasses/day	70	<5*		
	Female	<1 glass/week	0.5	28	0.70	0.40
		1–2 glasses/week	1.5	7		
		1–2 glasses/day	10.5	<5*		
		3–9 glasses/day	42	<5*		
		10+ glasses/day	70	<5*		
Smoking 1st and 2nd trimesters Alcohol 1st and 2nd trimesters	Male	<1 glass/week	0.5	63	1.97	5.63
		1–2 glasses/week	1.5	46		
		1–2 glasses/day	10.5	<5		
		3–9 glasses/day	42	<5		
		10+ glasses/day	70	<5*		
	Female	<1 glass/week	0.5	75	2.29	5.81
		1–2 glasses/week	1.5	34		
		1–2 glasses/day	10.5	10		
		3–9 glasses/day	42	<5		
		10+ glasses/day	70	<5*		

[1]Where alcohol had been categorised per day, the midpoint of the grouping was multiplied by 7 to estimate alcohol intake per week. Where alcohol intake was recorded as <1 glass/week, this was taken as 0.5 glasses/week.

[2] The mothers were asked to take "glass" to mean "a pub measure of spirits, half a pint of lager or cider, a glass of wine etc"

*This may include zero.

Table S5. Frequencies and mean alcohol levels (alcohol, 2nd trimester)[1].

Grouping	Sex	Alcohol Levels[2]	Mid Interval Value (m)	Frequency (f)	Mean (glasses/week)	Standard Deviation (glasses/week)
No smoking Alcohol 1st and 2nd trimesters	Male	<1 glass/week	0.5	466	1.22	3.42
		1–2 glasses/week	1.5	216		
		1–2 glasses/day	10.5	18		
		3–9 glasses/day	42	<5		
		10+ glasses/day	70	<5		
	Female	<1 glass/week	0.5	524	1.10	2.87
		1–2 glasses/week	1.5	234		
		1–2 glasses/day	10.5	16		
		3–9 glasses/day	42	<5*		
		10+ glasses/day	70	<5		
Smoking 1st trimester only Alcohol 1st and 2nd trimester	Male	<1 glass/week	0.5	27	0.84	0.47
		1–2 glasses/week	1.5	14		
		1–2 glasses/day	10.5	<5*		
		3–9 glasses/day	42	<5*		
		10+ glasses/day	70	<5*		
	Female	<1 glass/week	0.5	29	1.25	2.07
		1–2 glasses/week	1.5	13		
		1–2 glasses/day	10.5	<5		
		3–9 glasses/day	42	<5*		
		10+ glasses/day	70	<5*		
Smoking 1st and 2nd trimesters Alcohol 1st and 2nd trimesters	Male	<1 glass/week	0.5	70	1.20	1.83
		1–2 glasses/week	1.5	41		
		1–2 glasses/day	10.5	<5		
		3–9 glasses/day	42	<5*		
		10+ glasses/day	70	<5*		
	Female	<1 glass/week	0.5	81	1.28	2.15
		1–2 glasses/week	1.5	34		
		1–2 glasses/day	10.5	6		
		3–9 glasses/day	42	<5*		
		10+ glasses/day	70	<5*		

[1]Where alcohol had been categorised per day, the midpoint of the grouping was multiplied by 7 to estimate alcohol intake per week. Where alcohol intake was recorded as <1 glass/week, this was taken as 0.5 glasses/week.

[2] The mothers were asked to take "glass" to mean "a pub measure of spirits, half a pint of lager or cider, a glass of wine etc".

*This may contain zero.
